# Radiation shift from triple to quadruple frequency caused by the interaction of terahertz pulses with a nonlinear Kerr medium

**DOI:** 10.1038/s41598-022-13445-1

**Published:** 2022-05-30

**Authors:** Ilya Artser, Maksim Melnik, Azat Ismagilov, Mikhail Guselnikov, Anton Tcypkin, Sergei Kozlov

**Affiliations:** International Laboratory of Femtosecond Optics and Femtotechnologies, ITMO University, St. Petersburg, Russia

**Keywords:** Nonlinear optics, Terahertz optics

## Abstract

High-intensity optical radiation propagation in a transparent dielectric medium causes the phenomena of pulse self-action and radiation generation at triple frequencies due to the cubic nonlinearity of the medium. However, quadratic nonlinear effects usually outshine the cubic ones in anisotropic nonlinear crystals. In this work, we demonstrate that for certain experimental parameters the nonlinear effect of the third order can be stronger than the second order one in the MgO:$\hbox {LiNbO}_3$ crystal for terahertz frequency range. We experimentally and theoretically show that this effect can lead to the significant modification of the classical phenomenon of radiation generation at triple frequencies in the case when the pulse represents only one complete oscillation of the optical field. The experiment demonstrated that the phenomenon of generation of radiation at triple frequencies with respect to the frequency of the maximum spectral density in a nonlinear medium of the pulse disappears, and it is replaced by the generation of radiation at quadruple frequencies. The analysis confirms that this effect is based on the asymmetry and large width of the initial spectrum of such extremely short pulses in terms of the number of oscillations.

## Introduction

Research on the application of terahertz (THz) radiation in science and industry has been actively carried out over the past 30 years. THz radiation is attractive for such areas as the detection of hidden explosives, diagnostics of food and pharmaceutical products, diagnostics and therapy of socially significant diseases^1^, transmission and receiving of information, including wireless communication devices^2,3^, and various applications in space exploration^4^.

The very first source of electromagnetic THz radiation was created at the beginning of the last century by the Soviet scientist A.A. Glagoleva-Arkadyeva^5^. In recent years methods have been developed for efficient generation of high-intensity pulsed THz radiation, for example, the method of optical rectification^6^ or generation in plasma^7–9^. Under the excitation of high-intensity THz radiation, nonlinear effects of media become significant^10–12^. Remarkably, most sources of pulsed terahertz radiation generate pulses containing only a few oscillations of the electrical field^13^. Therefore, nonlinear optics of few-cycle pulses is one of the most perspective and actively developing scientific directions in the field of THz photonics.

Recently, it has been theoretically predicted and experimentally confirmed that the coefficient of the nonlinear refractive index of materials in the THz frequency range can be several orders of magnitude higher than its value for the same materials in the visible and near-IR spectral ranges^14–16^. This nonlinearity has a low-inertia mechanism; it means that high-speed THz photonics devices based on nonlinear effects are promising. The analysis of nonlinear effects in the field of pulsed THz radiation can be found in review papers^17–19^. In addition to the investigations of nonlinear phenomena features in the THz frequency range, new nonlinear materials for this range are actively studied and the existing ones are optimized to increase the efficiency of nonlinear processes for various applications. Currently, we can see advances in studies of the third harmonic generation of the THz frequency range in thin graphene layers^20,21^, as well as in doped semiconductors using unipolar THz pulses^22^.

Few-cycle THz pulses are characterized by interesting features of nonlinear optics such as a qualitative change in the nature of the familiar nonlinear effects. For example, for pulses with a small number of oscillations, the classical phenomenon of self-focusing may not be observed even when the critical self-focusing power exceeds many times^23^. Another classical phenomenon of generation of the second and third harmonics can be significantly modified in pulsed THz radiation, as shown in^24,25^.

For the first time we experimentally demonstrate that radiation generates at quadruple frequencies relative to the frequency of the power spectral density maximum due to the interaction of a THz pulse, which contains only one full oscillation of the electrical field, with a cubic nonlinear medium, while the expected radiation at triple frequency is absent. This effect may be determined by the asymmetry of the spectrum and large width of such a pulse and was analytically demonstrated in work^25^. In addition, based on theoretical calculations, the current work shows that in the THz frequency range, with a certain set of experimental parameters, it is possible to observe an excess of the cubic nonlinearity contribution over the quadratic one for the anisotropic nonlinear crystal; it is usually not observed in the visible frequency range. Therefore, within the framework of the work, it was possible to experimentally observe a pronounced cubic nonlinear effect in the anisotropic MgO:$\hbox {LiNbO}_3$ crystal. With refined analytical calculations, we demonstrate the dependence of dip position in the region of triple frequencies as well as its amplitude relation to the peak in the region of quadruple frequencies based on the nonlinear properties of the material. The analytical results obtained well agree with the experimental data. On the basis of this phenomenon, we have developed a method for evaluating the nonlinear refractive index.

## Results

### Experimental results

**Figure 1:**
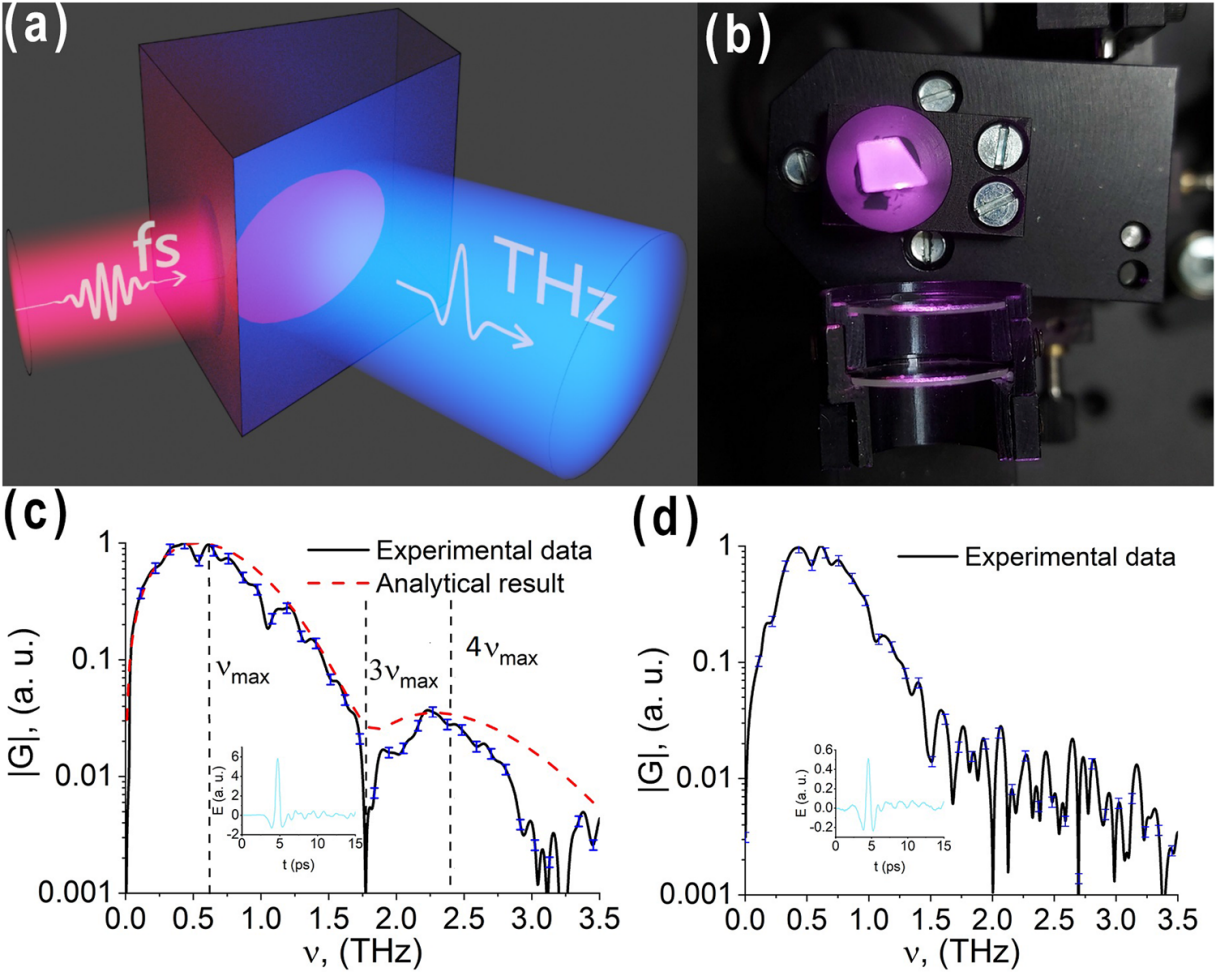
(**a**) Generation of the THz pulse by optical rectification. (**b**) Image of the generation of a THz pulse by the optical rectification method in MgO:$\hbox {LiNbO}_3$ crystal. (**c**) Spectrum of the generated THz field. The black solid line is the experimental data for high pump intensity, the red dotted line is the analytical results, the blue dashes show the experimental data error. (**d**) Spectrum of the generated THz field for lower pump intensity. Insets in (**c,d**) illustrate the temporal profile of the THz pulse at the output of the medium.

The phenomenon of new frequencies generation during the interaction of a high-intensity THz pulse with a nonlinear medium is experimentally observed using a scheme for THz pulse generation with the tilted wavefront in the MgO:$\hbox {LiNbO}_3$ crystal^26^. An image of the THz radiation generation by the optical rectification method is shown in Fig.1b.

Thus, a THz pulse is generated with an energy of up to 400 nJ and a duration of 1 ps. The diameter of the radiation beam at the crystal output is 2 mm, and the radiation intensity reaches 10$^8$ W/$\hbox {cm}^2$.

As seen in Fig.1c, no generation of radiation at triple frequencies ($3\nu _{max}$) is observed in the spectrum of pulsed THz radiation at the output of the medium during the interaction of the generated THz field with the crystal, which has cubic nonlinearity. Moreover, in the THz radiation spectrum instead of the triple frequency relative to the frequency of its spectral density maximum, a pronounced dip is observed. In this case, radiation of significant energy is generated at a quadruple frequency, which is not observed for similar experiments in the visible and near-IR frequency ranges. To explain this, the phenomenon has been theoretically studied.

Figure 1d shows the spectrum of a THz pulse generated at lower pump intensity. As seen, such low intensity is not enough to observe the nonlinear phenomenon of a new frequency generation. This confirms the fact that the phenomenon in Fig.1c, has purely nonlinear origin.

### Analytical results

**Figure 2:**
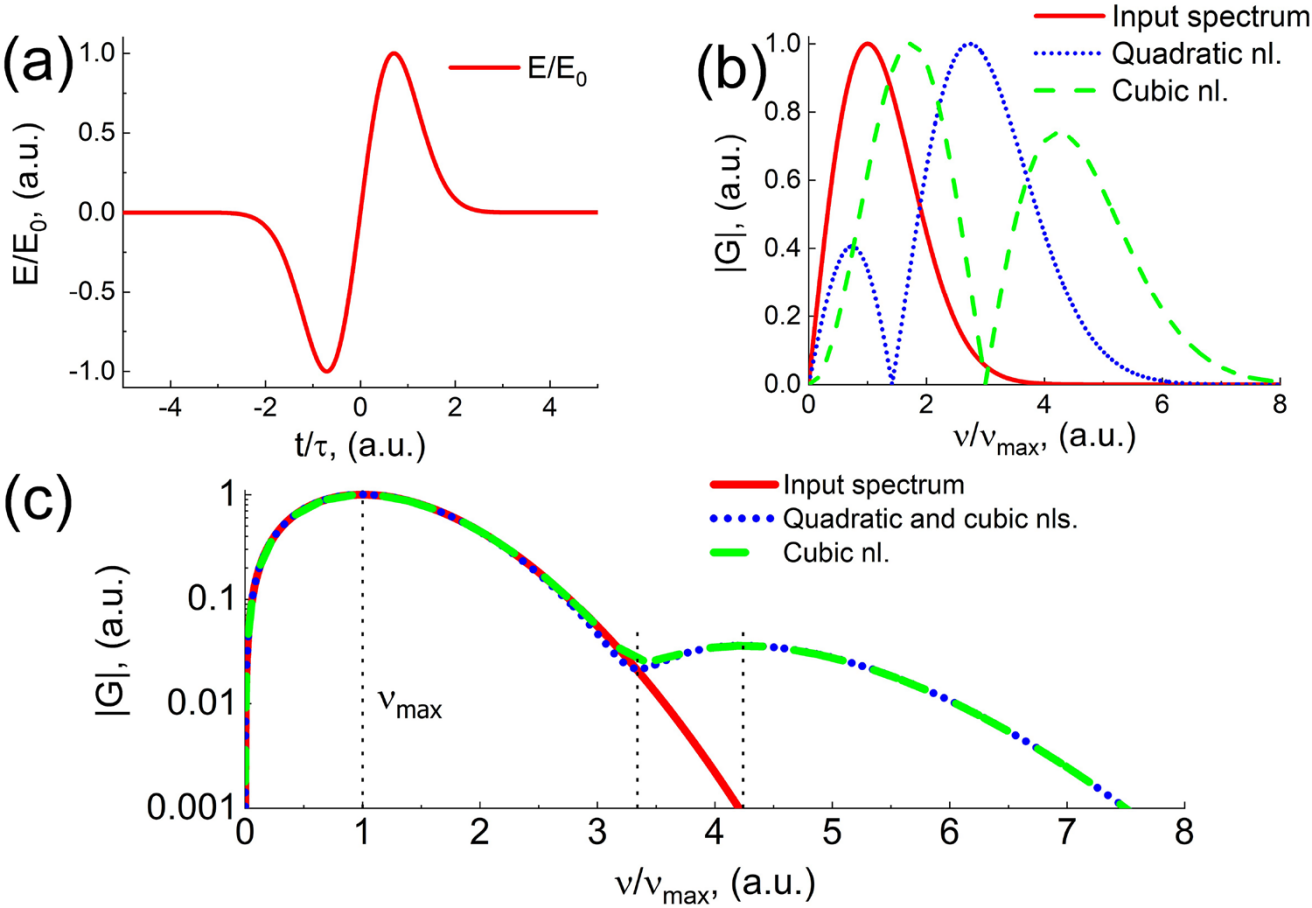
(**a**) Electric field of a THz single-cycle pulse (Eq. 2). (**b**) The THz pulse spectrum modulus at the input of the medium (the red curve, Eq. 10), modulus of second-order nonlinear contribution (the blue dotted curve, Eq. 12), modulus of third-order nonlinear contribution (the green dashed curve, Eq. 11). (**c**) Modulus of the THz pulse spectrum at the input of the medium (the red curve, Eq. 10) and the spectrum of the THz pulse at the output of the medium with (the blue dotted curve) and without (the green dashed curve) taking into account the second-order nonlinearity (Eq. 9). The position of the maximum frequency $\nu _{max}$ of the THz radiation is indicated.

On the basis of the proposed theoretical model (see Methods), the nonlinear interaction of THz radiation with a medium with quadratic and cubic nonlinearity was analytically studied; the results correspond to the experiment. The temporal dependence of the THz pulse field at the input of the optical medium (Eqs. 2, 4) is presented in Fig. 2a. Figure 2b demonstrates the spectrum modulus of such a THz pulse as well as the normalized modulus of changes in this spectrum due to quadratic and cubic nonlinearity of the medium (Eqs. 11, 12) in the THz frequency range. Figure 2c demonstrates the spectrum modulus of the THz pulse after its propagation in nonlinear medium (Eq. 9). The maximum of the spectrum modulus corresponds to the frequency of $\nu _{max}$ equal to 1 THz $(\tau =10^{-12}$ s). The equations parameters are chosen for the lithium niobate crystal used in the experiment: the THz radiation intensity $I=10^8$ W/cm$^2$, refractive index $N_0 =$ 5.15, the linear refractive index in the non-resonant electron contribution range $\hbox {n}_{el} = 2.26$, central frequency $\omega _0$ = 5.6 $\cdot 10^{12}$ s$^{-1}$, lattice constant $a_1 = 5.15 \cdot 10^{-8}$ cm, reduced mass of the vibrational mode $m = 1.5 \cdot 10^{-22}$ g, electric charge $q = 4.8 \cdot 10^{-10}$ Fr, thermal expansion coefficient $\alpha _T = 1.48 \cdot 10^{-5}$ K$^{-1}$, concentration $N=2.96 \cdot 10^{22}$ cm$^{-3}$, Boltzmann constant $k_B = 1.38 \cdot 10^{-16}$ erg $\cdot$ K$^{-1}$. All the parameters (their dependencies are shown in the figures) are normalized to their maximum values.

As seen from Fig. 2b, for a single-cycle pulse, the quadratic nonlinearity leads to the generation of tripled frequencies with respect to the maximum of the single-cycle pulse spectrum at the input of the nonlinear medium instead of the doubled ones, and the cubic nonlinearity leads to the generation of quadruple frequencies instead of the tripled ones. The first effect was described in^24^, and the second one in^25^. As seen from Fig. 2c, a pronounced dip is observed at the output of the nonlinear medium in the radiation spectrum in the region of tripled frequency with respect to the maximum spectral density of the initial pulse (the second dotted vertical line); it corresponds qualitatively to the experimental results (see Fig. 1c). Considering the medium with quadratic and cubic nonlinearity as well as with only cubic nonlinearity, radiation generates at higher frequencies with a maximum in the spectrum shifted to the region of quadruple frequencies of the maximum spectral density of a single-cycle pulse at the input of the nonlinear medium (the third dotted vertical line). The analytical expression for the spectrum at the output (Eq. 9) allows to estimate the position of the extrema of the function by taking derivative $\partial G/\partial \nu$ of Eq. (9). For example, for the parameters of the medium, as in Fig. 2c, the position of the minimum and maximum in the region of triple and quadruple frequencies is 3.34 and 4.24, respectively, and the ratio of their amplitudes is 2.14.

**Figure 3:**
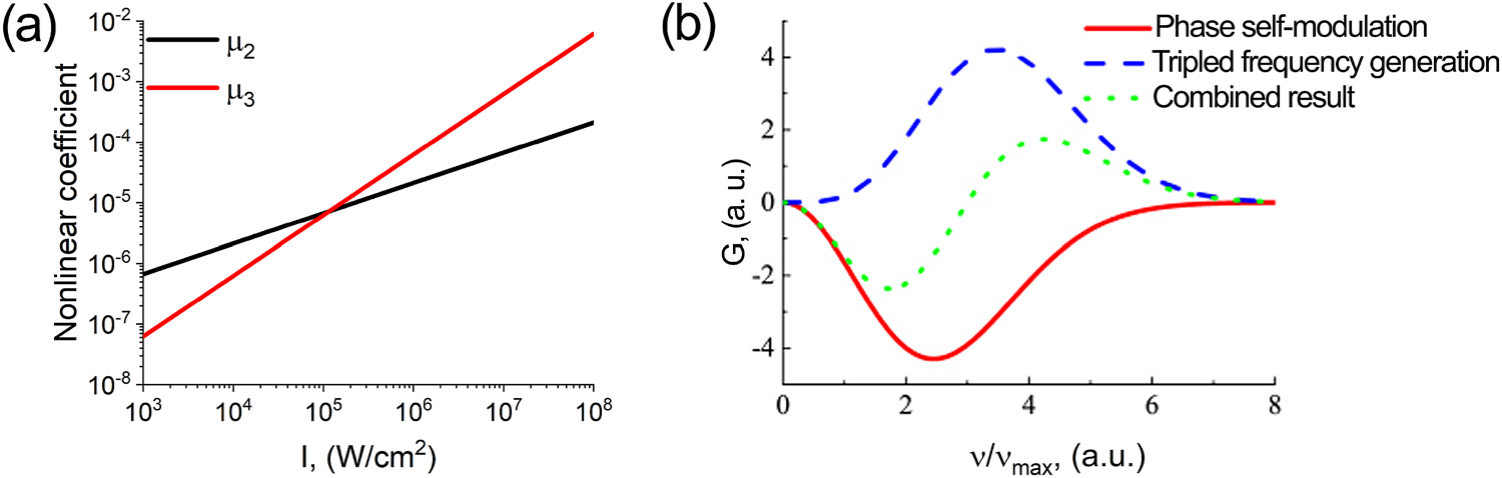
**(a)** Dependence of quadratic ($\mu _2$) and cubic ($\mu _3$) nonlinear coefficients on the THz pulse intensity. **(b)** Self-phase modulation (the solid red curve), tripled frequency generation (the dashed blue curve) contribution and combined result (the dotted green curve).

Notably, Fig. 2c demonstrates a negligible contribution of the quadratic nonlinearity with respect to the cubic one, which is not typical for anisotropic crystals in the optical range. To explain this phenomenon, we calculated the nonlinear coefficients corresponding to quadratic nonlinear contribution ($\mu _2$) and cubic contribution ($\mu _3$) from Eq. (3) (see “Methods”) depending on the radiation intensity. The results are presented in Fig. 3.

As seen from Fig. 3a, there is a range of intensities, at which the contribution of the quadratic nonlinearity is higher than that of the cubic one, as expected. However, in our experiment the intensity was 10$^8$ W/$\hbox {cm}^2$, and for this intensity the nonlinear coefficients can be calculated as $\chi _2=2.7 \cdot 10^{-6} {\mathrm{cm}}^{1/2} \cdot s \cdot {\mathrm{g}}^{-1/2}$, so that $\mu _2 = 2.11\cdot 10^{-4}$, while $\mu _3 = 6.21 \cdot 10^{-3}$. As clearly seen, the cubic nonlinearity contribution more than 30 times exceeds the quadratic one, which allows to observe the cubic nonlinear effect in the anisotropic crystal. This can be explained by the fact that in the THz range the value of nonlinear refractive index $\hbox {n}_2$ significantly exceeds the value for the visible and IR frequency ranges^16^. For lithium niobate, this excess is 4 orders of magnitude^15^.

We assume that such a paradoxical effect of shifting the position of the generated harmonic towards quadruple frequencies is caused by the pronounced asymmetry and large width of the single-cycle pulse spectrum (Fig. 2c, the red curve). The dip at tripled frequency is due to the interference of two parts of the nonlinear cubic contribution, self-phase modulation, and tripled frequency generation. Figure 3b illustrates these two contributions and their combined result. It can be seen that the contribution of self-modulation is negative, while the contribution of tripled frequency generation is positive. These nonlinear effects intersect and interfere due to the large width of the spectrum of a single-cycle pulse. Such interference results in a zero value of the total contribution, which coincides with the value of the tripled frequency.

Figure 2c shows the position of maximum frequency $\nu _{max}$ and triple and quadruple average frequency, where the average frequency is $\langle \nu \rangle =\int \limits _{-\infty }^{+\infty } \nu |G(\nu )| d\nu = 1.46 \nu _{max}$. As seen, the average frequency of the spectrum of a single-cycle pulse is slightly shifted relative to the maximum of the spectral density to the “blue” region. At high frequencies the position of the maximum in the radiation spectrum, which is generated in a medium with cubic nonlinearity, becomes equal to 3.3 relative to the average frequency.

**Figure 4:**
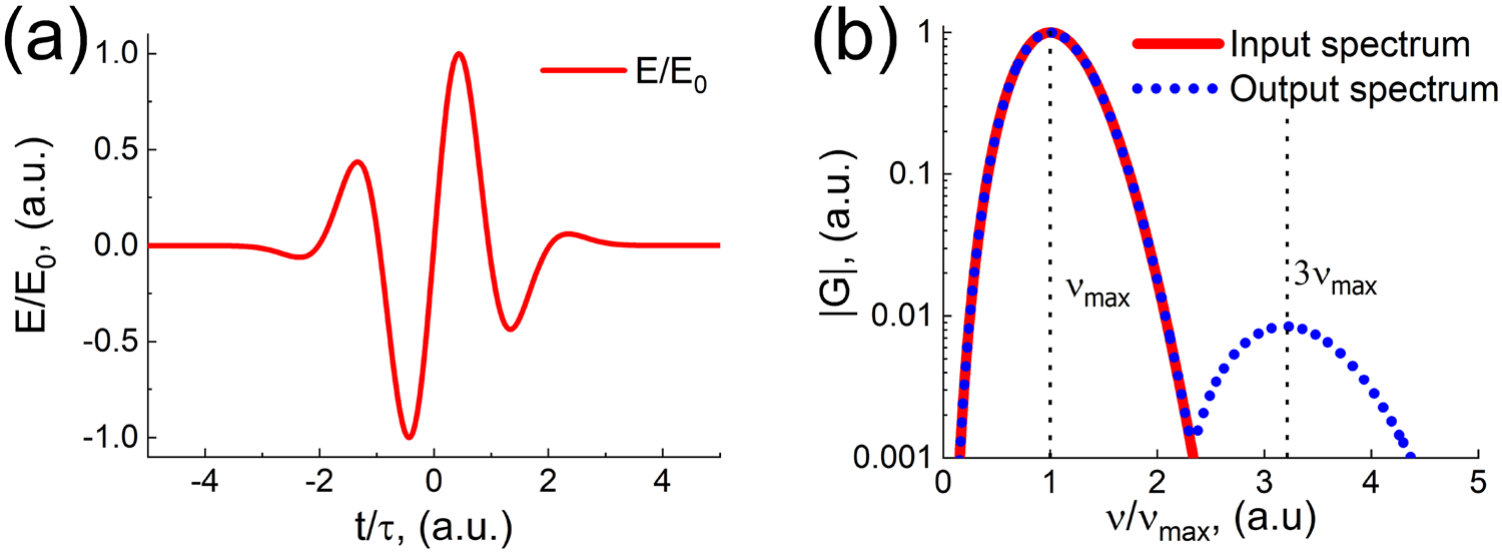
(**a**) Shape of the pulse with several oscillations electric field (Eq. 2). (**b**) Modulus of the pulse spectrum at the input of the medium (the red curve, Eq. 10) and the spectrum of the pulse at the output of the medium (the blue dotted curve, Eq. 9). The position of the maximum frequency $\nu _{max}$ of the THz radiation is indicated.

Nevertheless, with an increase in the number of periods in the pulse, the effect of the disappearance of radiation at tripled frequencies and the appearance of it at quadrupled ones vanishes. Figure 4 represents solutions (Eq. 9) considering only the cubic nonlinearity for a three-cycle pulse. It can be seen that even with such a small increase in the number of total field oscillations in the initial pulse, its spectrum becomes noticeably more symmetric and narrower. The effect of radiation generation at triple frequencies ($3\nu _{max}$) practically “returns”.

As a result of the theoretical discussion above, let us compare the results of the experiment and corresponding theoretical calculations. With the available characteristics of the crystal (which correspond to a crystal thickness of 0.5 cm) and the parameters of THz radiation $I=10^8$ ${\rm W/cm}^2$, $\tau =10^{-12}$ *s*, the experimental and theoretical curves are in close agreement, which is shown in Fig. 1c, and occurs for $n_2=(8\pm 1) \cdot 10^{-11}$ cm$^2/$W. This is the measured value of the nonlinear refractive index of the MgO:$\hbox {LiNbO}_3$ crystal in our work. The value is very close to the measurement result obtained by the authors of $n_2 = 4 \cdot 10^{-11}$ cm$^{2}$/W^27^. Note that in work^15^ the theoretical estimate of this coefficient was $5 \cdot 10^{-11}$ cm$^{2}$/W, which is in good good agreement with our measurement results. The difference in exact value is due to the assumptions and approximations of our theoretical model.

## Conclusion

We have experimentally shown for the first time that for the single-cycle THz pulse the phenomenon of radiation generation disappears at triple frequencies with respect to the frequency of the spectral density maximum in a nonlinear medium, and it is replaced by the generation of radiation at quadruple frequencies. Previously, this phenomenon was described analytically^25^. In our current work, we continued this study and derived analytical formulas (Eqs. 5–12) that allow us to predict the position of the dip in the region of triple frequencies and the peak in the region of the quadruple frequencies. We have also revealed the direct dependence of their ratio on the nonlinear properties of the medium. Furthermore, we have confirmed that this phenomenon is related to the strong asymmetry of the spectrum of a single-cycle pulse. In addition, it was found that in the THz frequency range, with a certain set of experimental parameters, it is possible to observe an excess of the cubic nonlinearity contribution over the quadratic one in a anisotropic lithium niobate crystal, which is usually not observed in the visible frequency range. This fact allows to observe a pronounced cubic nonlinear effect in MgO:$\hbox {LiNbO}_{3}$ crystal. We investigated the nonlinear effect and used it to measure the nonlinear refractive index of the MgO:$\hbox {LiNbO}_{3}$ crystal in the THz spectral range to be $n_2=(8\pm 1) \cdot 10^{-11}$ cm$^2$/W. This results obtained are in good agreement with other works.

## Methods

### Experimental setup

**Figure 5:**
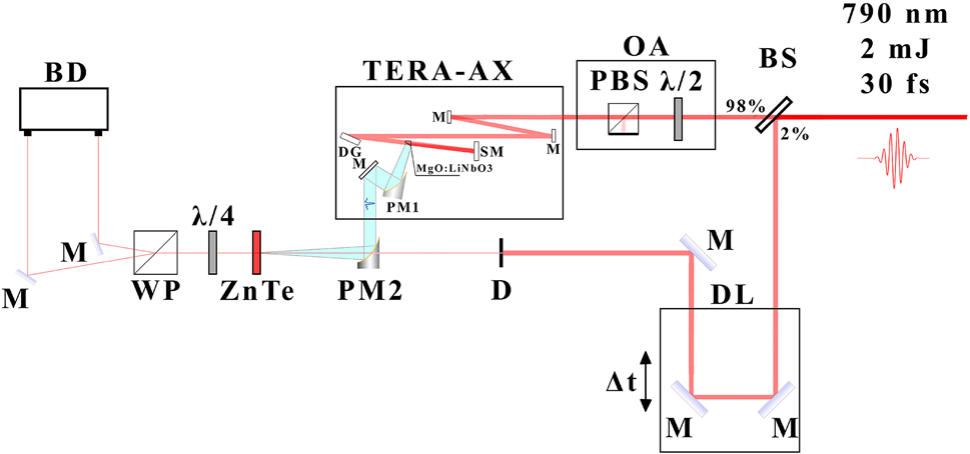
Schematic diagram of the experimental setup. Standard electro optical scheme for detecting the THz field using a 1 mm thick ZnTe crystal. The THz radiation source is a lithium niobate crystal, pumped by pulsed laser radiation. *BS* beamsplitter, *OA* optical attenuator, *PBS* poralizing beamsplitter, $\lambda$/2 half-wavelength plate, *TERA-AX* terahertz generator, *M* mirrors, *DG* diffraction grating, *SM* spherical mirror, *PM* parabolic mirror, *DL* delay line, *D* diaphragm, *ZnTe* 1 mm thick zinc telluride crystall, $\lambda$/4 quarter-wavelength plate, *WP* wollaston prism, *BD* balanced detector.

Experimental setup scheme is shown in Fig. 5. Radiation from a femtosecond laser system based on a regenerative amplifier (pulse duration is 30 fs, central wavelength is 790 nm, pulse energy is 2 mJ, repetition rate is 1 kHz) is divided into two beams with a beam splitter with 98:2 ratio. Pump radiation goes through an optical attenuator into TERA-AX THz generator. Pump radiation after the diffraction grating focused on MgO:$\hbox {LiNbO}_{3}$ crystal by the spherical mirror. Generated THz radiation collimated by the parabolic mirror and goes out of the system. After that THz radiation focused on 1 mm thick ZnTe crystal by another parabolic mirror for detection. After the beamsplitter the probe beam goes to the delay line and intersects with THz pulse at ZnTe crystal and is measured by the electro-optical setup.

Generation of THz radiation by the optical rectification method occurs in the entire volume of interaction of the tilted wavefront with the crystal. Thus, THz radiation is generated in the entire volume of crystal as well as during the pump radiation propagation through the medium with both quadratic nonlinearity, due to which the THz radiation generation process occurs, and cubic nonlinearity, due to which radiation is expected to be generated at triple frequencies with respect to the THz radiation. A schematic diagram of THz radiation generation is shown in Fig.1a. As a result, a diverging THz radiation beam with a Gaussian profile is formed, directed perpendicular to the crystal cut. Then, THz radiation is collimated using a parabolic mirror with a focal length of 25 mm. The THz field amplitude is measured using a standard electro-optical detection scheme in a 1 mm thick ZnTe crystal.

### Analytical model

The field approach allows to describe the dynamics of the THz radiation electric field in transparent dielectric medium. For instance, the equation which considers the inertialess quadratic and cubic nonlinearities as well as absorption and amplification can be represented as^28^:

1 $$\begin{aligned} \frac{\partial E}{\partial z}+\frac{N_0}{c}\frac{\partial E}{\partial t}-a\frac{\partial ^3 E}{\partial t^3}-(\Gamma -\gamma )E+ g_1 E\frac{\partial E}{\partial t} +g_2 E^2\frac{\partial E}{\partial t} =0, \end{aligned}$$

where E is electric field, z is the propagation direction, t is the time, *c* is the speed of light in vacuum, $\hbox {N}_0$ and *a* are the empirical constants characterizing the dependence of the linear refractive index of the medium on the frequency $\nu$ of the form $n(\nu )=N_0+4\pi ^2 ca\nu ^2$, $\Gamma$ is the amplification coefficient, $\gamma$ is the absorption coefficient, $g_1=\chi _2/c$ and $g_2=2n_2/c$, are the parameters characterizing the quadratic and cubic nonlinearity of the medium response respectively, $\chi _2$ is quadratic susceptibility and $n_2$ is the nonlinear refractive index coefficient of the medium (CGS units).

The field at the input of a nonlinear medium (at $z = 0$) is considered in the form of a single-cycle pulse (see Fig.2a) of the form:

2 $$\begin{aligned} E(t,0)=E_0\frac{t}{\tau }e^{-(\frac{t}{\tau })^2} \end{aligned}$$

where $E_0$ is the amplitude of the pulse electric field, $\tau$ is its duration. Figure 1a shows that the THz pulse observed in the experiment in the far diffraction zone has one and a half cycle. However, writing the boundary condition in the form of a single-cycle pulse (Eq. 2), we consider that during the propagation in the crystal, as well as at its output the THz pulse is a single-cycle one and only in the far zone, due to diffraction, it acquires a half-wave^29^. This effect, which is practically not observed for pulses with a large number of oscillations, is clearly pronounced for a single-cycle pulse, which becomes one and a half cycle in the far diffraction zone^30^.

For further analysis, it is useful to rewrite Eq. (1) in normalized variables ${\widetilde{E}}=E/E_0, {\widetilde{t}}=t/\tau , {\widetilde{z}}=z/L_{pulse}$, where $L_{pulse}=c\tau /N_0$ is the longitudinal size of the THz pulse in the medium:

3 $$\begin{aligned} \frac{\partial {\tilde{E}}}{\partial {\tilde{z}}}+\frac{\partial {\tilde{E}}}{\partial {\tilde{t}}}-\mu _0\frac{\partial ^3{\tilde{E}}}{\partial {\tilde{t}}^3}-\mu _1 {\tilde{E}}+\mu _2{\tilde{E}}\frac{\partial {\tilde{E}}}{\partial {\tilde{t}}}+\mu _3{\tilde{E}}^2\frac{\partial {\tilde{E}}}{\partial {\tilde{t}}} =0 \end{aligned}$$

Here, $\mu _0=1/2 \cdot \Delta n_{disp}/N_0$. $\Delta n_{disp}=4\pi ^2 ac \nu ^{2}_{max}$ characterizes the change in the refractive index due to dispersion, where $\nu _{max}=\sqrt{2}/\tau$ is the the spectral density maximum frequency at the input of the nonlinear medium, $\mu _1=\frac{c \tau }{N_0}(\Gamma -\gamma )$ characterizes the amplification and absorption of the medium; $\mu _2=\frac{\chi _{2} E_{0}}{N_0}$ represents the quadratic nonlinearity contribution, where $\chi _{2}=\frac{m \omega _{0}^2 a_{l} \alpha _{T} }{32 \pi ^2 q N k_{B}}(n_{0,\nu }^2-1)^2$, $\mu _3=4\Delta n_{nl}/N_0$, $\Delta n_{nl}=1/2 n_2 E^{2}_{0}=n^{'}_{2}I$ characterizes the change in the refractive index of the medium due to the cubic nonlinearity, $n^{'}_{2}$ is the coefficient of the nonlinear refractive index of the medium (in SI units), *I* is intensity of THz pulse, $n_{0,\nu }^2 = \sqrt{1+N_0^2 - n_{el}^2}$ is the vibrational contribution to the low-frequency refractive index, $n_{el}$ is the refractive index in the range with nonresonant electronic contribution (800 nm), $\omega _0$ is the fundamental vibration frequency, $a_1$ is the lattice constant, *m* is the reduced mass of the vibrational mode, *q* is the effective charge of the chemical bond, $\alpha _T$ is the thermal expansion coefficient, *N* is the number density of vibrational units and $k_B$ is the Boltzmann constant.

The normalized boundary condition (Eq. 2) takes the form

4 $$\begin{aligned} {\widetilde{E}}({\widetilde{t}},0)={\widetilde{t}}e^{-{\widetilde{t}}^2} \end{aligned}$$

The contribution of dispersion, as known, leads to a change in the temporal structure of the pulse and does not affect its spectrum. In addition, it can be seen from the experimental data that no dispersive spreading of the pulse is observed; therefore, the dispersive contribution can be neglected.

The dependence of the absorption spectrum in the studied spectral range has no characteristic features^31^. The result of taking into account the absorption coefficient leads to a monotonic decrease in the power spectrum of the output THz radiation. In this regard, absorption can be neglected in calculations.

For ease of taking into account the amplification of the THz pulse, in the calculations we consider the case when the energy of the pulse interacting with the medium corresponds to the energy of the pulse at the output from the medium. Therefore, in the propagation dynamics equation, the amplification term can also be omitted.

Thus, for an analytical calculation of the dynamics of a THz pulse during propagation, one can focus on the last two terms of Eq. (3). Assuming the values $\mu _2$ and $\mu _3$ are small, the solution to Eq. (3) can be found in the form of a series:

5 $$\begin{aligned} {\tilde{E}}\left( {\tilde{t}},{\tilde{z}}\right) \,=\,{\tilde{E}}^0\left( {\tilde{t}},{\tilde{z}}\right) +\mu _2{\tilde{E}}^{(1,\text {nl2})}\left( {\tilde{t}},{\tilde{z}}\right) +\mu _3{\tilde{E}}^{(1,\text {nl3})}\left( {\tilde{t}},{\tilde{z}}\right) \end{aligned}$$

It is easy to show that with boundary conditions (Eq. 2), the terms in such a solution take the form

6 $$\begin{aligned} {\widetilde{E}}^{(0)}({\widetilde{t}},{\widetilde{z}})\,=\, & {} ({\widetilde{t}}-{\widetilde{z}})e^{-({\widetilde{t}}-{\widetilde{z}})^2} \end{aligned}$$

7 $$\begin{aligned} {\tilde{E}}^{(1,\text {nl2})}\left( {\tilde{t}},{\tilde{z}}\right)\,=\, & {} {\widetilde{z}} ({\widetilde{t}} - {\widetilde{z}}) (2({\widetilde{t}} - {\widetilde{z}})^2-1) e^{-2({\widetilde{t}} - {\widetilde{z}})^2} \end{aligned}$$

8 $$\begin{aligned} {\widetilde{E}}^{(1,nl3)}({\widetilde{t}},{\widetilde{z}})\,=\, & {} {\widetilde{z}} ({\widetilde{t}} - {\widetilde{z}})^2 (2({\widetilde{t}} - {\widetilde{z}})^2-1) e^{-3({\widetilde{t}} - {\widetilde{z}})^2} \end{aligned}$$

Here, ${\widetilde{E}}^{(0)}$ is the solution of Eq. (3) for the “zero” approximation, i.e. without taking into account the nonlinearity of the medium, ${\widetilde{E}}^{(1,nl2)}$ and and ${\widetilde{E}}^{(1,nl3)}$ are the change in the shape of the pulse, associated with quadratic and cubic nonlinearity respectively.

Accordingly, the field (Eq. 5) spectrum $G({\widetilde{\nu }}, {\widetilde{z}})=1/\sqrt{2\pi }\int \limits _{-\infty }^{+\infty } {\widetilde{E}}({\widetilde{t}},{\widetilde{z}}) \cdot e^{i{\widetilde{\nu }}{\widetilde{t}}} d{\widetilde{t}}$ where ${\widetilde{\nu }}=\nu \tau$ can be represented as

9 $$\begin{aligned} {\widetilde{G}}({\widetilde{\nu }},{\widetilde{z}})\,=\, & {} {\widetilde{G}}^{(0)}({\widetilde{\nu }},{\widetilde{z}})+\mu _2 {\widetilde{G}}^{(1,nl2)}({\widetilde{\nu }},{\widetilde{z}})+\mu _3 {\widetilde{G}}^{(1,nl3)}({\widetilde{\nu }},{\widetilde{z}}) \end{aligned}$$

10 $$\begin{aligned} {\widetilde{G}}^{(0)}({\widetilde{\nu }},{\widetilde{z}})\,=\, & {} \frac{i {\widetilde{\nu }}}{2\sqrt{2}}e ^ {i {\tilde{z}} {\tilde{\nu }} - {\widetilde{\nu }}^2/4} \end{aligned}$$

11 $$\begin{aligned} G^{(1,\text {nl2})}\left( {\tilde{\nu }},{\tilde{z}}\right)\,=\, & {} -\frac{i {\tilde{\nu }} {\tilde{z}} ({\widetilde{\nu }}^2 - 4)}{64}e^{-{\tilde{\nu }}^2/8 + i {\tilde{\nu }} {\tilde{z}}} \end{aligned}$$

12 $$\begin{aligned} {\widetilde{G}}^{(1,nl3)}({\widetilde{\nu }},{\widetilde{z}})\,=\, & {} \frac{{\tilde{z}} {\tilde{\nu }}^2 ({\widetilde{\nu }}^2 - 18)}{648 \sqrt{6}}e^{-{\widetilde{\nu }}^2 / 12 + i {\tilde{\nu }} {\tilde{z}}} \end{aligned}$$

## Data Availability

All data generated or analyzed during this study are included in this published article and its supplementary information files.
